# Respiratory microbiome and clinical course of carbapenem-resistant *Acinetobacter baumannii* pneumonia in critically Ill patients

**DOI:** 10.1097/MD.0000000000038988

**Published:** 2024-08-02

**Authors:** Jin Gu Yoon, Sooyeon Lim, Hak-Jun Hyun, Hye Seong, Ji Yun Noh, Joon Young Song, Woo Joo Kim, Hee Jin Cheong

**Affiliations:** a Division of Infectious Diseases, Department of Internal Medicine, Korea University College of Medicine, Seoul, Republic of Korea; b Asia Pacific Influenza Institute, Korea University College of Medicine, Seoul, Republic of Korea; c Vaccine Innovation Center-KU Medicine, Seoul, Republic of Korea.

**Keywords:** *Acinetobacter baumannii*, intensive care units, microbiota, pneumonia

## Abstract

Carbapenem-resistant *Acinetobacter baumannii* (CRAB) pneumonia has been a serious problem in the intensive care unit (ICU). However, defined characteristics of respiratory microbiome in CRAB pneumonia are lacking nowadays. This study aimed to analyze respiratory microbiome of CRAB pneumonia compared to non-CRAB pneumonia and reveal the clinical significance of respiratory microbiome data in these patients. Patients diagnosed with severe pneumonia with mechanical ventilation were enrolled in the ICU of a tertiary care hospital. Respiratory specimens were collected on days 1, 4, 7, and 14 in each participant via tracheal aspiration. Clinical data and outcomes of each enrolled patient were collected via electronic medical records. Microbiome analysis was conducted with collected respiratory specimens undergone by next-generation sequencing of microbial 16S ribosomal DNA. Six CRAB pneumonia, 4 non-CRAB pneumonia and 5 healthy controls were enrolled. In CRAB pneumonia, CRAB was detected in 3 patients by sputum culture at day 1, while it was negative at day 1 and detected later in the others by follow-up sputum culture. Beta diversity plot analysis showed differences between each group. Shannon index was decreased markedly at day 4 in CRAB pneumonia compared to the others. Among CRAB pneumonia cases, 3 respiratory specimens were culture-negative, but positive by microbiome analysis. Lower respiratory microbiome in CRAB pneumonia had distinct characteristics and early loss of diversity compared to non-CRAB pneumonia, which might be related to poor clinical course. Moreover, CRAB acquisition and colonization would be predicted by preemptive microbiome analysis, which will contribute to effective infection control in the ICU.

## 1. Introduction

Bacterial pneumonia caused by *Acinetobacter baumannii* is associated with high mortality and morbidity, especially in critically ill patients admitted to intensive care units (ICUs).^[1]^ The genus *Acinetobacter* is an aerobic Gram-negative, non-fermentative nonpigmented and oxidase-positive or negative coccobacilli. They frequently found in wet environments including soil, sludge, and seawater, containing various antibiotic resistance genes such as carbapenemase and extended-spectrum β-lactamases. Human skin and mucous membrane including respiratory tract are also well-known site of colonization.^[2]^ Among *Acinetobacter* species, *A baumannii, Acinetobacter pittii, Acinetobacter nosocomialis, Acinetobacter seifertii*, and *Acinetobacter dijkshoorniae* were associated with human diseases.^[3]^ Despite its nonpathogenic colonization, *A baumannii* harbors diverse virulence factors that play roles in biofilm formation, adherence, quorum sensing, immune evasion and cellular cytotoxicity, contributing to the development of critical infections.^[4,5]^

The clinical burden of *A baumannii* infections has increased considerably in parallel with the era of advanced intensive care and global antimicrobial resistance.^[6]^ Carbapenem-resistant *A baumannii* (CRAB) was declared as a major concern of health care-associated infection by World Health Organization in 2017.^[7]^ In Korea, imipenem resistance of *A baumannii* had been increasing, reaching 85% in 2015.^[8]^ Another important point is an easy person-to-person transmission, resulting in nosocomial outbreak events of CRAB. *A baumannii* can be transferred to patients via close contact with healthcare workers and nearby contaminated environments in ICUs.^[9]^ As CRAB usually has multiple resistant mechanisms to widely used antibiotics, a standard treatment for CRAB infection has not been established. Combination regimens with carbapenem, polymyxin, aminoglycosides, sulbactam, minocycline, tigecycline and novel β-lactam/β-lactamase inhibitors have been evaluated for the treatment of CRAB infections. However, the overall patient prognosis is poor.^[10]^

Interest in respiratory microbiomes is increasing in both the clinical and laboratory settings. The traditional concept of respiratory tract is that it is sterile and requires cleaning to treat the respiratory infections. However, many microorganisms reside and interact with each other in the respiratory tract. The normal microbiome of the lower respiratory tract, mainly consists of anaerobes, including *Prevotella, Firmicutes, Proteobacteria, Veillonella, Fusobacterium, Streptococcus*, and *Pseudomonas* genera. The normal respiratory microbiome is associated with diet, geographic region, culture and nearby microbiomes, such as those in the oral cavity and upper gastrointestinal tract.^[11]^ The normal respiratory microbiome serves as a gatekeeper that prevents the growth of potential pathogens.^[12]^ In contrast, oral streptococci, anaerobes and common pathogens such as *S pneumoniae* and *H influenzae* are more frequently detected in the bronchoalveolar lavage fluid of patients with pneumonia.^[13]^ Given the development of 16S ribosomal DNA sequencing, which amplifies a relatively small amount of bacterial DNA found in respiratory specimens, correlations between the respiratory microbiome and chronic lung diseases, such as asthma, cystic fibrosis, and chronic obstructive pulmonary disease (COPD), have been revealed in recent studies.^[11,14,15]^ Similarly, several clinical and laboratory studies have identified an association between respiratory dysbiosis and hospital-acquired pneumonia.^[16]^

However, the colonization and infection of the respiratory tract by multidrug-resistant microorganisms and their interactions with the respiratory microbiome remain unclear. Our study aimed to analyze the respiratory microbiome of critically ill patients with CRAB pneumonia compared with those without CRAB pneumonia in ICU settings. In addition, we aimed to clarify the timing of CRAB acquisition after admission and its relationship with respiratory dysbiosis based on microbiome analysis.

## 2. Methods

### 2.1. Study population and enrollment

This prospective observational was conducted at Korea University Guro Hospital, Seoul, Korea. The center is a tertiary care hospital that operates medical, emergency, and surgical ICUs. Adult patients aged > 18 years, who were diagnosed with severe pneumonia and received mechanical ventilation during ICU admission, were enrolled. Pneumonia was defined and classified as community- or hospital-acquired based on the Infectious Disease Society of America/American Thoracic Society guideline and 2008 Centers for Disease Control and Prevention/National Healthcare Safety Network criteria, according to radiologic evidence (2 or more serial chest radiographs with at least one of the following: new or progressive and persistent infiltrate, consolidation, and cavitation), signs and symptoms (at least one of the following: fever, leukopenia (<4000 WBC/mm^3^) or leukocytosis (≥12,000 WBC/mm^3^), altered mental status for individuals ≥ 70-years-old, and at least one of the following: new onset of purulent sputum, new onset cough or dyspnea/tachypnea, rales, and worsening gas exchange), and laboratory findings (at least one of the following: microbiologic culture evidence, and histopathological examination).^[17–19]^ The airway was secured with intubation or tracheostomy tubes. Informed consent was obtained from the patients or their immediate family members before enrollment. The exclusion criteria were as follows; immunocompromised patients prescribed cytotoxic chemotherapy within the previous 6 months, >20 mg/day of systemic steroids (prednisolone base) or use of other immunosuppressants, history of organ transplantation, or human immunodeficiency virus infection, and patients who refused or were unable to obtain informed consent. CRAB was defined as *A baumannii* that was resistant to imipenem and meropenem. The enrolled participants were divided into the CRAB pneumonia group (one or more CRAB detections in tracheal aspirate cultures during the study period) and the control (CRAB was never detected in tracheal aspirate cultures during the study period) group. Furthermore, the CRAB pneumonia group was divided into a day 1 positive group (assumed to already carry CRAB), and day 1 negative group (assumed to have acquired CRAB infection after enrollment). Healthy control participants were enrolled to compare the healthy and diseased microbiomes.

### 2.2. Clinical data collection

Clinical data for each patient were collected from the electronic medical records. The baseline characteristics included demographic data, history of antibiotics use, aspiration events, tracheostomy status, functional activity (bedridden or ambulatory), feeding route (oral or tube feeding), vasopressor use, type of nebulizer, length of hospitalization, and mortality. The Charlson comorbidity index was calculated for all participants to validate multiple underlying illnesses, including myocardial infarction, heart failure, peripheral vascular disease, cerebrovascular accident, dementia, COPD, connective tissue disease, peptic ulcer disease, liver disease, diabetes, hemiplegia, chronic kidney disease, solid tumor, hematologic malignancy and acquired immunodeficiency syndrome.^[20]^ Antimicrobial susceptibility profiles of CRAB were also collected, which was determined by Clinical & Laboratory Standards Institute (CLSI) guidelines.

### 2.3. Specimen collection

We defined “day 1” as the day of the pneumonia diagnosis. For each ICU enrolled patient, 5–10ml of respiratory specimens were collected at day 1, 4, 7, and 14 via tracheal aspiration. We also collected sputum from healthy control at once. Standard sputum Gram stain and culture were performed simultaneously using the same specimen collection route. Seegene Allplex^TM^ respiratory panel assays (Seegene Inc., Seoul, Republic of Korea) were used to detect bacteria (*Bordetella pertussis, Chlamydophila pneumoniae, H influenzae, S pneumoniae, Legionella pneumophila,* and *Mycoplasma pneumoniae*) and viruses (adenovirus, enterovirus, metapneumovirus, parainfluenza virus, influenza virus, respiratory syncytial virus, bocavirus, seasonal coronavirus, and rhinovirus) by real-time polymerase chain reaction (PCR) on day 1. Streptococcal urinary antigen test (Alere BinaxNOW^®^ *S. pneumoniae* antigen card) and blood culture were also performed at the same day. Microbial species identification and antimicrobial susceptibility tests were taken by VITEK II automated system (BioMérieux Inc., Marcy-l’Etoile, France), utilizing a standard identification card and broth dilution method. Susceptibility tests were interpreted according CLSI guidelines.^[21]^ The specimen for microbiome analysis was transported by REST^TM^ NB SWAB, which was instantly frozen in −80°C or temporarily refrigerated in 4°C up to 48 hours before frozen.

### 2.4. Bacterial community analysis

Hypervariable V3 and V4 regions were selected for amplicon sequencing of the 16s rRNA gene.^[22,23]^ DNA was extracted using the HostZERO^TM^ Microbial DNA kit (ZYMO RESEARCH, CA). Library preparation for 16S metagenomic sequencing was performed according to the manufacturer protocol (Illumina). Briefly, 16S sequences were amplified in the 1st-round PCR using Phusion high-fidelity DNA polymerase (ThermoFisher, Waltham, MA) and 16S amplicon PCR primer pairs (Cosmogenetech, Daejeon, South Korea): forward 5’-TCG TCG GCA GCG TCA GAT GTG TAT AAG AGA CAG CCT ACG GGN GGC WGC AG-3’; reverse 5’-GTC TCG TGG GCT CGG AGA TGT GTA TAA GAG ACA GGA CTA CHV GGG TAT CTA ATC C- 3’ and the following thermocycling program: 95°C for 3 minutes, 95°C for 30 seconds, 55°C for 30 seconds, and 72°C for 30 seconds for 25 cycles; further, 72°C for 5 minutes and held at 4°C indefinitely. After PCR clean-up, Nextera dual indices (Illumina, San Diego, CA) were attached in the 2nd-round PCR using Nextera Index Primers N7XX and S5XX and the following program: 95°C for 3 minutes, 95°C for 30 seconds, 55°C for 30 seconds, and 72°C for 30 seconds for 8 cycles, followed by 72°C for 5 minutes, and held at 4°C indefinitely. After 2nd PCR clean-up, the library was validated using a 2100 Bioanalyzer (Agilent, Santa Clara, CA, USA) and quantified by RT-qPCR (KAPA). After pooling, the library was denatured and diluted to the final concentration of 8 pm The phiX control was included in the library.

The metagenomics workflow classifies bacteria from a metagenomic sample by amplifying specific regions of 16S ribosomal RNA. The reads were classified using the Greengene taxonomy database of 16S ribosomal RNA data. The metagenomics workflow multiplexes index reads, generates FASTQ files, and classifies the reads at the taxonomic level.

The richness and diversity of samples were determined by operational taxonomic units (OTUs) and diversity indices (Shannon diversity indices). The samples were filtered based on the relative abundance of each identified OTU. Relative abundance was calculated based on the MiSeq read data. Bacterial community profiles of sample sequence read with > 1% relative abundance. The metagenome profile included counts of reads aligned to each contig, which were analyzed using metagenomic tools and R.^[24]^ Heatmap and correlation plot analyses were conducted to determine the beta diversity. Conversely, the Shannon diversity index was calculated to compare alpha diversity between the groups.^[25]^ Principal component analysis (PCA) of variance was conducted to identify the best set of variables to describe community structure using a permutational multivariate analysis of variance (PERMANOVA) test. Statistical analyses were performed using R v.4.1.2 (R Foundation for Statistical Computing, Vienna, Austria).

### 2.5. Ethical statements

The study was conducted in accordance with the Declaration of Helsinki, and approved by the Institutional Review Board of Korea University Guro Hospital (approval No. 2020GR0104, approval date 2020-03-05). Informed consents were obtained from all participated patients or immediate family members.

## 3. Results

### 3.1. Patient characteristics

**Figure 1.**
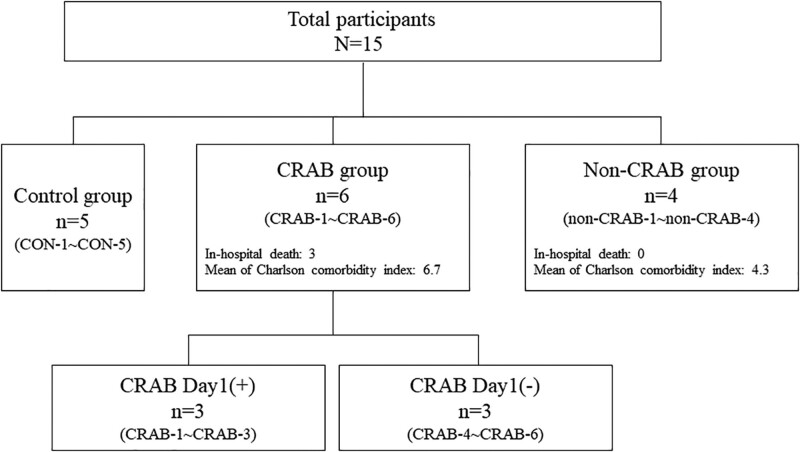
Participant enrollment and grouping: Fifteen participants were enrolled, with 6 grouped as having CRAB pneumonia, 4 grouped as having non-CRAB pneumonia, and 5 as healthy controls. CRAB: carbapenem-resistant *Acinetobacter baumannii*

Fifteen participants were enrolled in the study including 5 healthy controls. Six patients were grouped as having CRAB pneumonia and had already carried CRAB at admission (day 1 positive: n = 3) or had acquired during pneumonia treatment (day 1 negative: n = 3). CRAB was never detected in 4 patients during treatment; therefore, they were grouped as having non-CRAB pneumonia. Three of the 6 CRAB patients died from CRAB pneumonia, with a higher mean Charlson comorbidity index (6.7 in CRAB group vs 4.3 in non-CRAB group) (Fig. 1 and Table 1). Five healthy controls (2 males aged 27 and 64 years and 3 females aged 42, 45, and 66 years) did not have any underlying illness or antibiotic history.

**Table 1:** Baseline characteristics of patients with CRAB and non-CRAB pneumonia in ICUs (N = 10).

	CRAB pneumonia						Non-CRAB pneumonia			
	CRAB-1	CRAB-2	CRAB-3	CRAB-4	CRAB-5	CRAB-6	Non-CRAB-1	Non-CRAB-2	Non-CRAB-3	Non-CRAB-4
Age	78	59	88	72	83	73	71	67	77	73
Sex	M	M	M	M	M	F	F	M	M	F
Duration of hospitalization (d)	17	22	23	45	24	13	41	8	19	29
Length of ICU stay (d)	17	10	12	5	12	13	4	4	19	29
Maintenance of tracheostomy	N	Y	N	Y	N	N	Y	N	Y	Done in hospitalization (HD#24)
Initial diagnosis	HAP	HAP	HAP	HAP	HAP	CAP	HAP	CAP	HAP	CAP
Aspiration event history	N	N	N	N	Y	N	N	N	N	N
Antibiotics history within 3 mo	Y	Y	Y	N	Y	N	Y	N	N	N
Bedridden	Y	Y	N	Y	Y	N	Y	N	Y	Y
Nutrition	TPN, Tube feeding	Tube (PEG) feeding	Tube feeding	Tube feeding	Tube feeding	Tube feeding	Tube feeding	Oral feeding	Tube feeding	Tube feeding
Inotropics	Y	N	N	N	Y	Y	N	N	Y	N
Nebulizer	Acetylcysteine	N	Acetylcysteine	Acetylcysteine	N	N	N	Ipratropi-um, Salbuta-mol	N	Acetylcysteine
Underlying disease	Stroke	Brain hemorrh-age	Dementia	DM, COPD	Stroke	ICMP, LC, Stroke	Brain hemorrhage	COPD	Brain hemorrhage, Parkinson disease, Alzheimer disease	DM, Alzheimer disease
Charlson comorbidity index	6	2	6	6	8	12	4	3	5	5
Respiratory bacterial panel	*H influenzae*	Negative	Not done	Negative	Negative	Not done	*S pneumoniae*	*H influenzae*	*H influenzae*	*S pneumoniae*
Respiratory virus PCR panel	Negative	Negative	Not done	Negative	Negative	Not done	Negative	Negative	Negative	Negative
Streptococcus urinary antigen test	Not done	Not done	Negative	Negative	Negative	Not done	Positive	Negative	Negative	Positive
Specimen d 1 Culture	CRAB	CRAB	CRAB	Normal flora	Normal flora	Normal flora	Normal flora	Normal flora	Normal flora	Normal flora
Specimen d 4 Culture	CRAB	Normal flora	CRAB	CRAB	CRAB	CRAB	*P aeruginosa*	Normal flora	Normal flora	Normal flora
Specimen d 7 Culture	CRAB	CRAB	Not done	CRAB	Not done	CRAB	*P aeruginosa*	Normal flora	*P aeruginosa, S pneumoniae*	Normal flora
Specimen day 14 culture	Normal flora	Not done	CRAB	Normal flora	Normal flora	Not done (lost)	*P aeruginosa*	Discharged	*P aeruginosa*	Normal flora
Blood culture	CRAB, *S aureus, E faecallis, K pneumonia, S agalactiae*	Not detected	Not detected	*S cerevisiae, S epidermidis*	Not detected	*S aureus*	Not detected	Not detected	Not detected	Not detected
Mortality	Y	N	N	N	Y	Y	N	N	N	N

M, male; F, female; Y, yes; N, no; CAP, community-acquired pneumonia; HAP, hospital-acquired pneumonia; HD, hospital day; TPN, total parenteral nutrition; PEG, percutaneous endoscopic gastrostomy; DM, diabetes mellitus; COPD, chronic obstructive pulmonary disease; ICMP, ischemic cardiomyopathy; LC, liver cirrhosis; ICU, intensive care unit; CRAB, carbapenem-resistant *Acinetobacter baumannii*.

The baseline characteristics of the enrolled patients are shown in Table 1. Respiratory specimens were collected from all patients on days 1, 4, 7, and 14, with the exception of those who were discharged or died before day 14. The CRAB pneumonia group had more comorbidities and a higher mortality rate (50.0% vs 0%) than the non-CRAB pneumonia group. Two patients in the CRAB pneumonia group could not undergo multiplex PCR testing due to patient refusal. The CRAB pneumonia group used anti-pseudomonal broad-spectrum antibiotics in combination more frequently than in the non-CRAB pneumonia group (83.3% vs 50.0%) (Fig. S1–2) http://links.lww.com/MD/N242. The antimicrobial susceptibility pattern of CRAB cases was similar in all patients, with high minimal inhibitory concentrations (MICs) of imipenem (≥16 mg/L), meropenem (≥16 mg/L), ampicillin-sulbactam (≥32 mg/L) and gentamicin (≥16 mg/L). Patient CRAB-2 had different susceptibilities to ampicillin-sulbactam (=8mg/L) and remained resistant. All CRAB strains were susceptible to colistin (≤0.5 mg/L).

### 3.2. Bacterial community analysis

Microbiome analyses were performed on all respiratory specimens except for the day 14 sample of patient non-CRAB-1, which was lost due to contamination, and day 7 and 14 samples of patient CRAB-5, in which the amount of tracheal aspirate samples was insufficient. A quality check of PCR via bioanalysis quantification showed that the appropriate size of the amplified products for the V3-V4 regions of 16S ribosomal DNA sequences was approximately 550 bp in all specimens.

**Figure 2.**
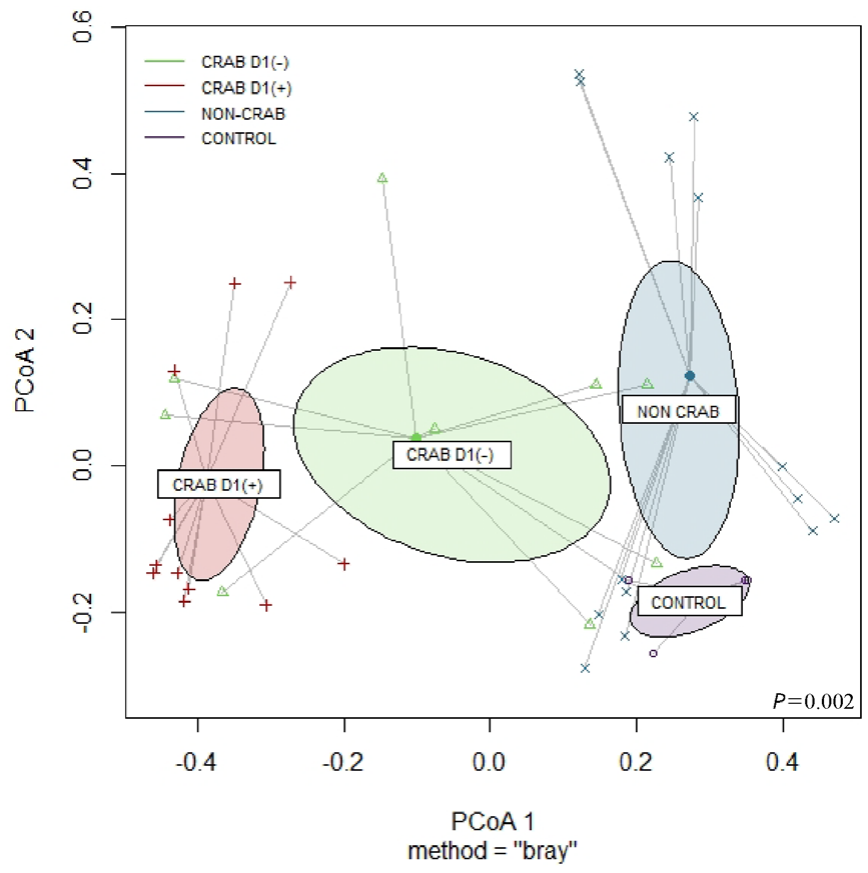
Beta diversity based on each group, principal coordinate analysis (PCoA) using Bray–Curtis distances. The distance between points represents differences in microbiome community composition. The ellipses represent 95% confidence intervals of sample groups. Microbiome samples were significantly differed by the groups (*P* = .002). Average distances to median values are as follows: CONTROL: 0.5519, CRAB D1(−): 0.5631, CRAB D1(+): 0.3263, NON-CRAB: 0.5855. Value of PCoA1: 4.0020, PCoA2: 2.2446. D1(−), d 1 negative; D1(+), d 1 positive.

The PCA using Bray–Curtis distances was performed to analyze beta diversity in each group, which revealed significant intergroup differences (*P* = .002; Fig. 2) of microbial composition. Based on the non-CRAB pneumonia group and healthy controls, the early-stage CRAB pneumonia group (day 1 positive) had a more significant distance than the latter group (day 1 negative), which means that the composition of microbiome was more different. Additional bootstrapping was done for assigning the accuracy of sample estimate and enhancing the measurement (Fig. S3). http://links.lww.com/MD/N242 We did not consider antibiotic use in this analysis because the classes and durations of antibiotic use were diverse. A beta diversity analysis comparing each group of specimens is shown in Supplementary Figures 3 and 4. http://links.lww.com/MD/N242 In the correlation plot analysis (Fig. S4), http://links.lww.com/MD/N242 thinner and bluer diagonal lines indicated a greater correlation between the microbial composition of each sample. This suggests a close correlation between samples of CRAB-1 to 4 and relatively low correlation between patients of CRAB-1, 4, 5, and 6 and patients of non-CRAB-1 to 4. A heatmap with phylogenetic analysis (Fig. S5) http://links.lww.com/MD/N242 indicated the relationship of patients CRAB-1 to 4 with *A. baumannii* and *A gerneri*. Patients non-CRAB-1 to 3 were related to *Streptococcus pseudopneumoniae* and *Streptococcus tigurinus*.

**Figure 3.**
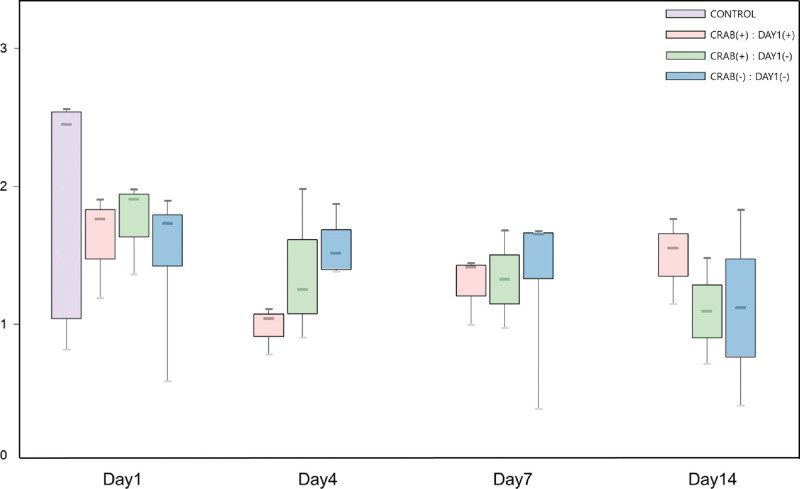
Shannon index of CRAB pneumonia group (d 1 positive includes CRAB-1 to -3 and d 1 negative includes CRAB-4 to -6), non-CRAB pneumonia group, and healthy control group. The box range represents first and third quartiles. Lines indicate high, median and low values. Note that an early significant decreasing index score was observed in the CRAB pneumonia d 1 positive and d 1 negative groups.

**Figure 4.**
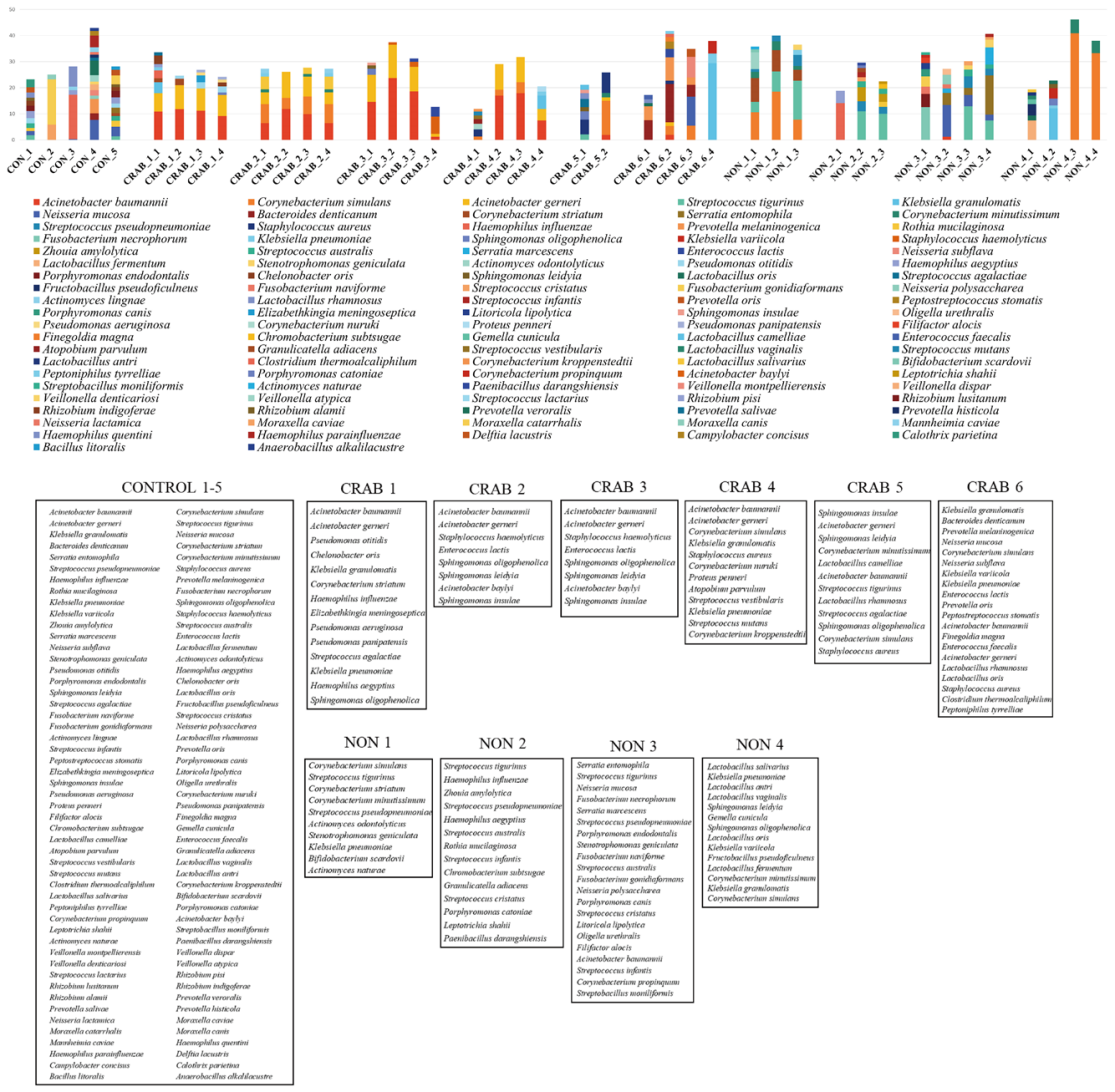
Serial changes of microbial compositions in all patients. CRAB-1 to-6 indicates cases of CRAB-1 to -6 in the CRAB pneumonia group. Conversely, NON-1 to -4 indicates non-CRAB-1 to -4 in the non-CRAB pneumonia group. CON-1 to -5 indicates healthy controls. Each sample end number (-1 to -4) means the time sequence in which samples were taken (days 1, 4, 7, and 14). Each box contains the name of the species, without order. Note that patients CRAB-5 and -6 were dominated by *Acinetobacter* species, *Corynebacterium simulans, Klebsiella granulomatis, Neisseria mucosa,* and *Staphylococcus aureus*.

The Shannon index of each group exhibited an early decrease in the index score in the CRAB pneumonia group for both day 1 positive and negative subgroups, suggesting a loss of species diversity during the early stage of CRAB pneumonia, regardless of CRAB colonization or acquisition (Fig. 3). In cases in which CRAB was initially detected on day 1, suggesting colonization or infection by CRAB before enrollment, microbiome analysis revealed the dominance of *A baumannii* and *A gerneri*. The proportion of *Acinetobacter* species decreased in CRAB-2 and 3 patients, who were treated with meropenem and ampicillin-sulbactam (Figs. 4 and S1). http://links.lww.com/MD/N242 The CRAB pneumonia group detected after day 1, which suggests CRAB were acquired during hospitalization, showed a dominance of *A baumanni, A gerneri, Corynebacterium simulans, Klebsiella granulomatis* and *S aureus* (Fig. 4). The non-CRAB pneumonia group was dominated by *C simulans, Corynebacterium striatum, S pseudopneumoniae, S tigurinus, Neisseria mucosa* and *H influenzae* (Fig. 4). The 16S ribosomal DNA of *A baumannii* was detected in one sample on day 4 from patient non-CRAB-3, in whom CRAB was not detected in tracheal aspirate cultures during the study period. Nearly half of the sequences could not be classified at species level.

### 3.3. Correlation of respiratory microbiome with sputum culture and multiplex PCR

The detection of *Acinetobacter* species in tracheal aspirate cultures and microbiome analyses are shown in Supplementary Table 1. http://links.lww.com/MD/N242 *Acinetobacter* species were detected in 3 samples by microbiome analysis but not by culture analysis. In contrast, *Acinetobacter* species were detected by culture analysis in one sample but not by microbiome analysis. In the case of *Pseudomonas* species, all 5 samples from non-CRAB-1 and -3 patients were tracheal aspirate culture-positive. However, these were not detected by the microbiome analysis (Table S2). http://links.lww.com/MD/N242

*S. pneumoniae* was detected by multiplex PCR in 2 patients (non-CRAB-1 and -4). However, *S pneumoniae* was not detected in either patient in the microbiome analysis. Only *S pseudopneumoniae* were detected in the non-CRAB-1 patient. *H influenzae* was detected in 3 patients (CRAB-1, non-CRAB-2, and -3) by multiplex PCR and in 2 patients (CRAB-1 and non-CRAB-2) by microbiome analysis (Table 1).

## 4. Discussion

The respiratory microbiome of CRAB pneumonia group was distinguished from those of the non-CRAB pneumonia and healthy control groups using beta diversity analysis. Furthermore, the CRAB pneumonia group exhibited an early decrease in alpha diversity compared to the non-CRAB pneumonia group, suggesting a loss of diversity during the early stages of CRAB pneumonia, regardless of CRAB carriage before admission or during hospitalization.

Several factors affect the respiratory microbiome, including intubation, feeding, probiotics, antibiotics, underlying diseases, and specific microorganisms.^[11,26,27]^ Additionally, critical illness in patients and the unique environment of the ICU may cause changes in the respiratory microbiome.^[28]^ These confounding factors may complicate evaluation of the effects of a single constituent. Furthermore, information regarding the causal relationship between a single microorganism and the entire microbial niche remain scarce, which suggests their complex interconnections.

Our study describes the relationship between CRAB pneumonia and loss of microbiome diversity. Different microbiome compositions between the CRAB and non-CRAB pneumonia groups were observed using beta diversity analysis. Moreover, microbiome diversity, determined by species richness and evenness using alpha diversity analysis, significantly decreased after day 1 in the CRAB group, especially in the carrier (day 1 positive) group. The proportion of CRAB in the microbiome was high for each CRAB-positive sample. These results suggest that the respiratory microbiome in CRAB pneumonia has a distinct composition and is related to a rapid drop in the diversity index compared with that in non-CRAB pneumonia. However, whether CRAB causes changes in diversity, or vice versa, remains unclear. In addition, the difference between true CRAB infections and colonization remains unclear. The high prevalence of comorbidities, disease severity, and broad-spectrum antibiotic use in the CRAB group may have affected the results. Nevertheless, our conclusions suggest the importance of CRAB in the respiratory microbiome, and invite a larger prospective cohort study involving patients in the ICU to clarify the mechanisms and pathogenesis of CRAB pneumonia. Molecular analyses are required to evaluate the relationships among the respiratory microbiome, inflammatory cytokines and antibiotic resistance.

Most microbiomes in CRAB pneumonia were dominated by *A baumannii* and *A gerneri*. Actually, 16S ribosomal DNA sequencing uses only approximately 550 bp of the V3-V4 region to identify microorganisms at the species level, with > 97% correlation between sequences. Therefore, the species of microorganisms present may not be fully defined by 16S ribosomal DNA sequencing compared with whole-genome sequencing. A similar phenomenon was observed in *S pneumoniae, S pseudopneumoiae, S tigurinus, C simulans*, and *C striatum*. In microbiome analysis, *S pseudopneumoniae* is a group of viridans streptococci that is frequently misidentified as *S pneumoniae. S pseudopneumoniae* may also act as a pathogen. SP2020 is a useful marker for distinguishing *S pneumoniae* and may contribute to the investigation of the precise composition of Streptococcus in future microbiome studies.^[29,30]^

The correlation between microbiome analysis and clinical tracheal aspirate cultures or multiplex PCR indicates the diagnostic value of microbiome analysis. Three cases of *Acinetobacter* species were detected using microbiome analysis alone; therefore, this method may be an early detector of CRAB acquisition. Note that *A baumannii* was detected by microbiome analysis of a sample from non-CRAB pneumonia group (non-CRAB-3). This might result from specimen contamination during endotracheal suction because the abundance was relatively low (1.147%) and no *A baumannii* was detected in subsequent samples. Therefore, the false positive of microbiome analysis should be considered when detecting CRAB. Moreover, high costs and prolonged examination times are hurdles that need to be overcome. In contrast to *Acinetobacter* species, *P aeruginosa* was better detected using the culture method. This can be explained by the degradation of Pseudomonas DNA during pretreatment.^[31]^ The possibility of contamination by non-glucose fermenters such as *Acinetobacter* and *Pseudomonas* species via water sources should be considered.

This study has certain limitations. First, the sample size is small. Large-scale studies are required to determine the robust statistical significance of differences in microbiomes. Second, the subgroup classification into day 1 positive and day 1 negative in the CRAB pneumonia group may not indicate carriage or acquisition. A false-negative result for CRAB detection by microbiome analysis on day 1 may have missed a CRAB carriage. The selection pressure of broad-spectrum antibiotics might have contributed to the increase in the CRAB population in the microbiomes after day 1 and should be considered when interpreting the results. Third, we independently collected tracheal aspirate cultures microbiome specimens, which may have affected the differences between the culture and microbiome analyses results. Fourth, a relatively high proportion of unspecified species (up to 50%) was observed for each specimen. This may be due to the large proportion of host DNA in endotracheal aspirates or degradation of bacterial DNA during the extraction phase. Therefore, enhanced microbial DNA selection is necessary for further studies. Fifth, broad-spectrum antibiotics were used more frequently in the CRAB pneumonia group, which may have contributed to the lower diversity scores. Carbapenem exposure during the early stages affected the microbiome of the CRAB pneumonia day 1 positive group. However, the Shannon index was restored despite continuous carbapenem use, suggesting that antimicrobials were not the only factors responsible for the observed microbiome changes. A prospective, controlled study is necessary to exclude the confounding effects of antibiotic use. Nevertheless, our results suggest specific characteristics of the respiratory microbiome in CRAB pneumonia, which may be used as markers of CRAB acquisition and colonization or as markers of clinical prognosis.

In conclusion, the lower respiratory microbiome in CRAB pneumonia has distinct characteristics and an early loss of diversity compared with that in non-CRAB pneumonia, which was related to a poor clinical course. Furthermore, CRAB acquisition and colonization can be predicted using preemptive microbiome analysis, which can contribute to effective infection control in ICUs. Overall, the microbiomes can be used as markers for the diagnosis and prognosis of CRAB pneumonia. Owing to the small sample size in our study, larger prospective molecular studies are necessary to investigate causal relationships and build useful microbiome markers.

## Author contributions

**Conceptualization:** Jin Gu Yoon, Joon Young Song, Hee Jin Cheong.

**Data curation:** Sooyeon Lim.

**Formal analysis:** Sooyeon Lim.

**Funding acquisition:** Jin Gu Yoon, Sooyeon Lim, Hee Jin Cheong.

**Methodology:** Sooyeon Lim.

**Resources:** Jin Gu Yoon.

**Software:** Jin Gu Yoon, Sooyeon Lim.

**Validation:** Jin Gu Yoon, Sooyeon Lim.

**Writing – original draft:** Jin Gu Yoon, Sooyeon Lim, Hye Seong.

**Writing – review & editing:** Hak-Jun Hyun, Ji Yun Noh, Joon Young Song, Woo Joo Kim, Hee Jin Cheong.

## Supplementary Material


