# Supplementary material for: Respiratory microbiome and clinical course of carbapenem-resistant Acinetobacter baumannii pneumonia in critically Ill patients
Source: Medicine (Baltimore). 2024 Aug 2;103(31):e38988. doi: 10.1097/MD.0000000000038988 (PMC11296444; doi:10.1097/MD.0000000000038988)

**Supplementary Table 1.** Detection of Acinetobacter species in CRAB pneumonia group by microbiome analysis and tracheal aspirate culture

|  | Day 1 | Day 4 | Day 7 | Day 14 |
| --- | --- | --- | --- | --- |
| CRAB-1 | ● | ● | ● | ◎ |
| CRAB-2 | ● | ◎ | ● | N/A |
| CRAB-3 | ● | ● | N/A | ● |
| CRAB-4 | X | ● | ● | ◎ |
| CRAB-5 | X | ● | N/A | N/A |
| CRAB-6 | X | ● | ○ | N/A |

●, Detected by both methods; ◎, Detected by microbiome analysis only; ○, Detected by culture only; X, Not detected by both methods; N/A, one of both methods not available

**Supplementary Table 2.** Detection of Pseudomonas species by microbiome analysis and tracheal aspirate culture

|  | Day 1 | Day 4 | Day 7 | Day 14 |
| --- | --- | --- | --- | --- |
| CRAB-1 | X | ◎ | ◎ | ◎ |
| Non-CRAB-1 | ○ | ○ | ○ | X |
| Non-CRAB-3 | X | ○ | ○ | X |

●, Detected by both methods; ◎, Detected by microbiome analysis only; ○, Detected by culture only; X, Not detected by both methods


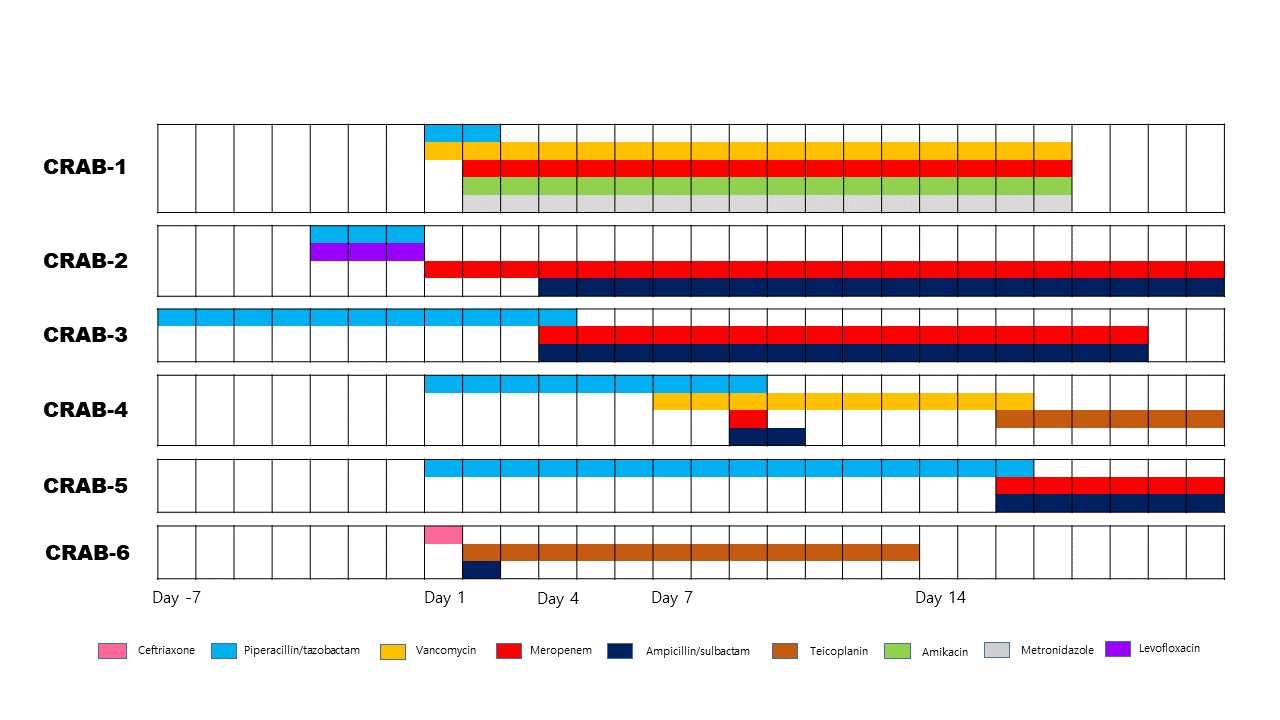


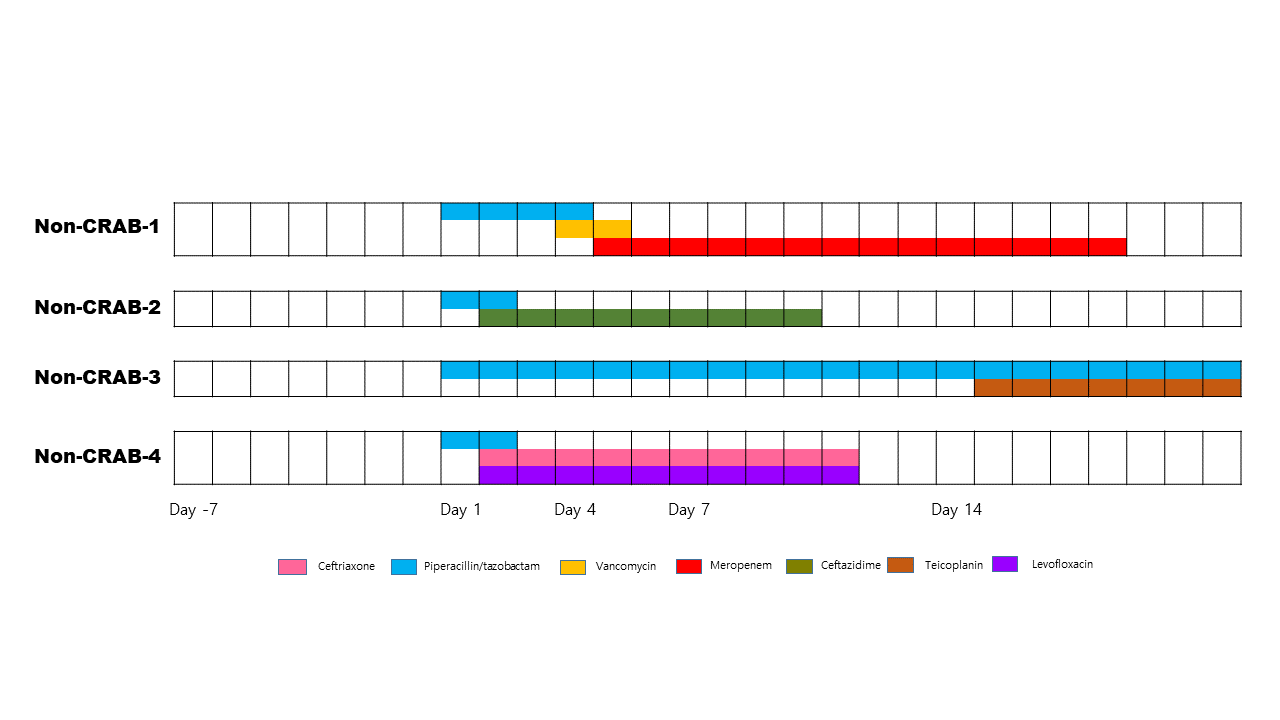


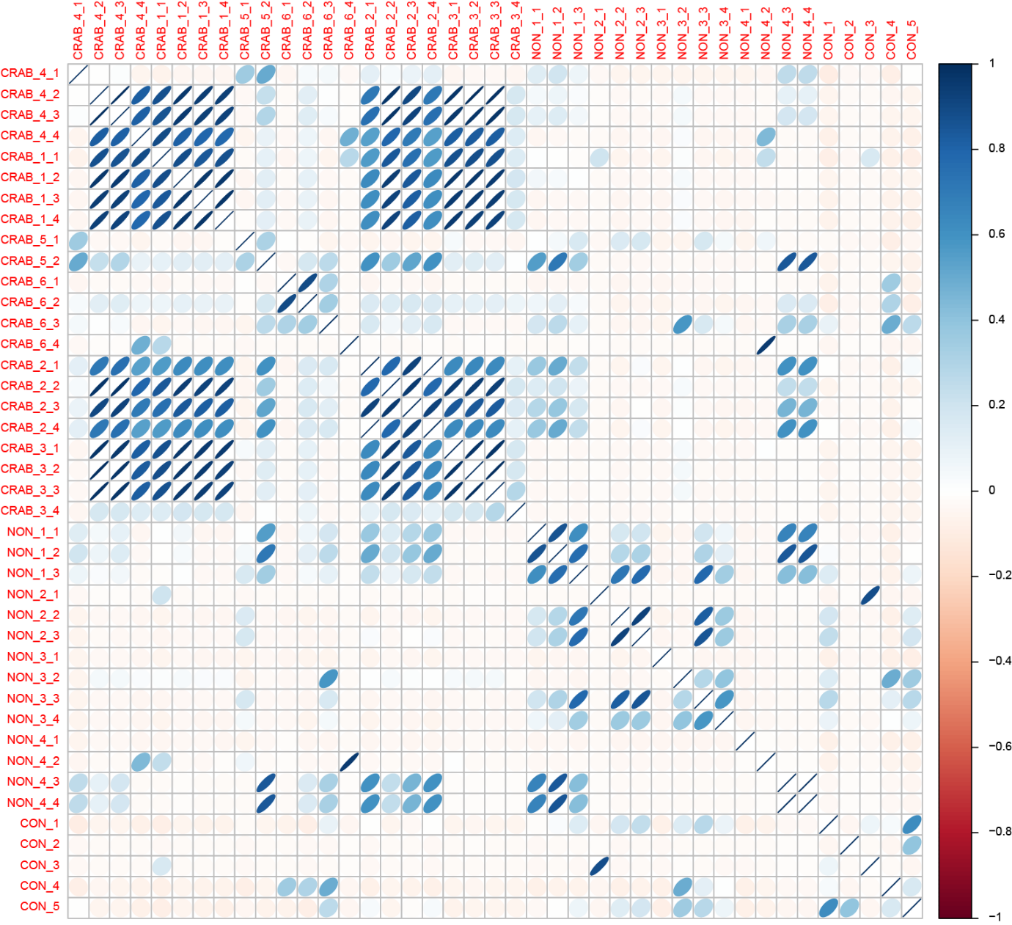


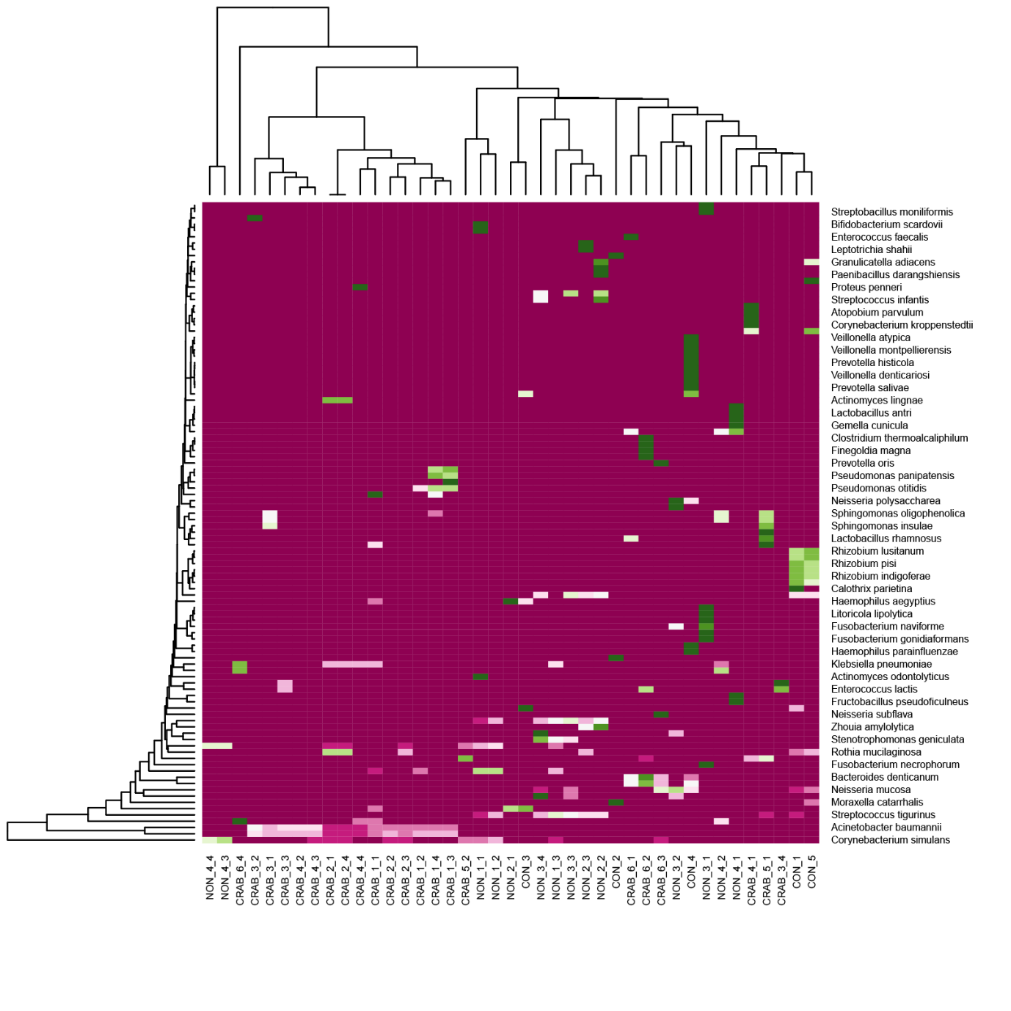

Supplement: Supplementary file 1 [file medi-103-e38988-s001.docx]
